# Supplementary material for: Haptophyte-infecting viruses change the genome condensing proteins of dinoflagellates
Source: Commun Biol. 2025 Mar 28;8:510. doi: 10.1038/s42003-025-07905-3 (PMC11953307; doi:10.1038/s42003-025-07905-3)
Supplement: Supplementary file 1 — Supplementary information [file 42003_2025_7905_MOESM1_ESM.pdf]

Supplementary information

Haptophyte-infecting viruses change the genome condensing proteins of dinoflagellates

Haina Wang, Lingjie Meng, Sara Otaegi-Ugartemendia, Gabriela Nérida Condezo, Romain Blanc-Mathieu, Runar Stokke, Marius Rydningen Langvad, David Brandt, Jörn Kalinowski, Håkon Dahle, Carmen San Martín, Hiroyuki Ogata, Ruth-Anne Sandaa

Supplementary Figure 1. Phylogenetic tree of marine NCLDVs constructed by protein sequences of DNA polymerase B

Supplementary Figure 2. Phylogenetic tree aligned with sequences alignment and NLS prediction

Supplementary Figure 3. Structural prediction and pairwise sequence alignment by Alphafold2.0, coloured by pLDDT confidence measure

Supplementary Table 1. Summary of posttranslational modifications encoded by HeV RF02 and PkV RF02.

Supplementary Table 2. Core genes of the flagellated-protist-infecting viruses

Supplementary Table 3. Acronyms

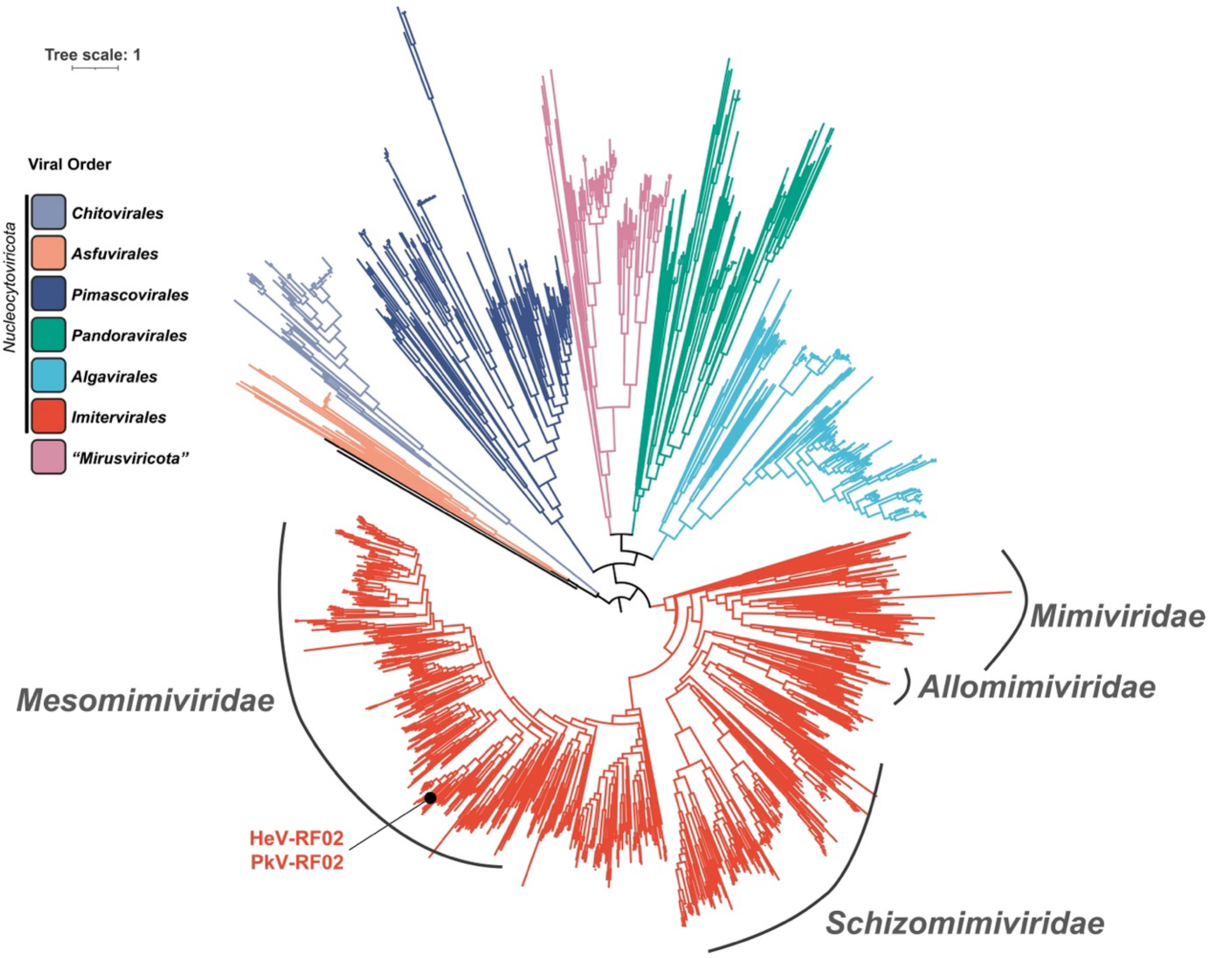

Supplementary Figure 1. Phylogenetic tree of marine NCLDVs constructed by protein sequences of DNA polymerase B. The positions of HeV RF02 and PkV RF02 and their relative viruses are noted. Colour ranges of different taxonomy classifications are listed in the left-hand panel.

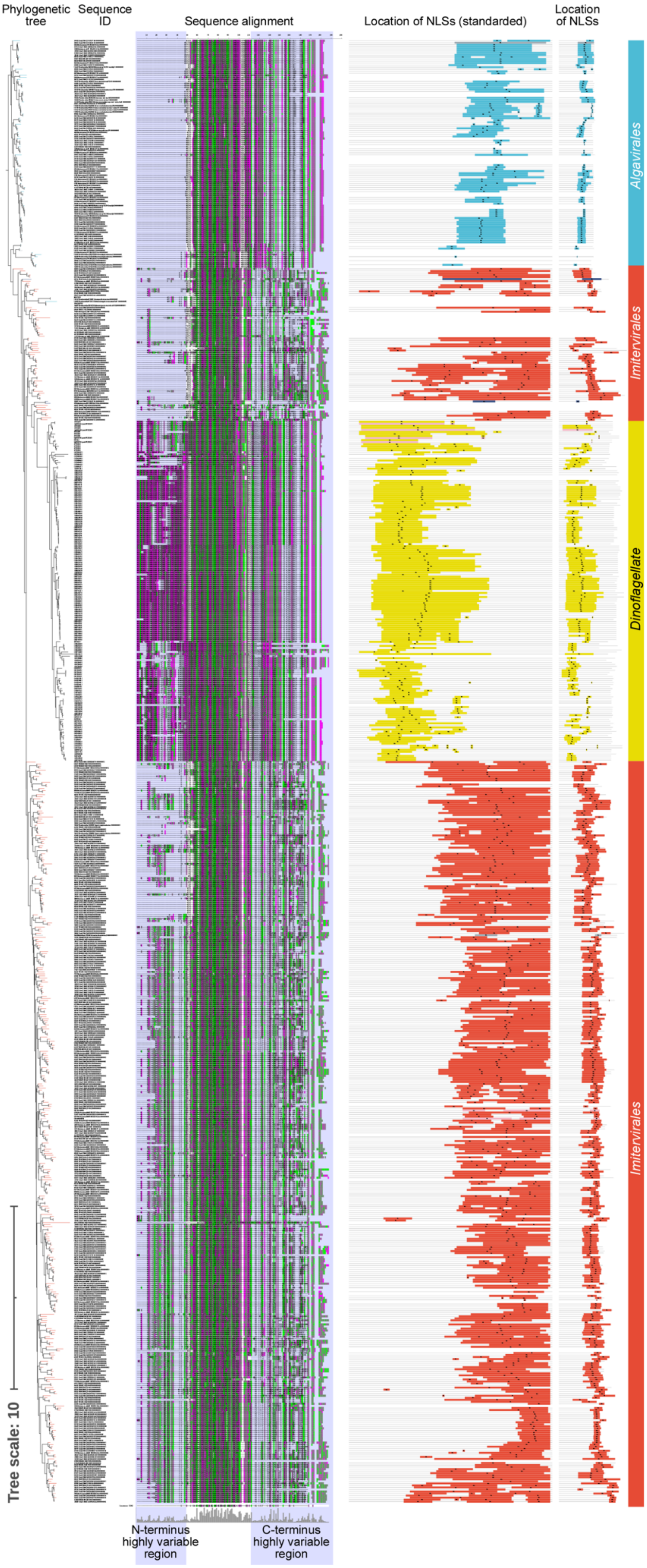

Supplementary Figure 2. Phylogenetic tree aligned with sequences alignment and NLS prediction. The phylogenetic tree is the same one as Fig. 4B with the expanded dinoflagellate DVNPs clade. The predicted NLSs are highlighted by different colours to show their classification. The highly variable regions in the sequence are indicated by the light blue areas.

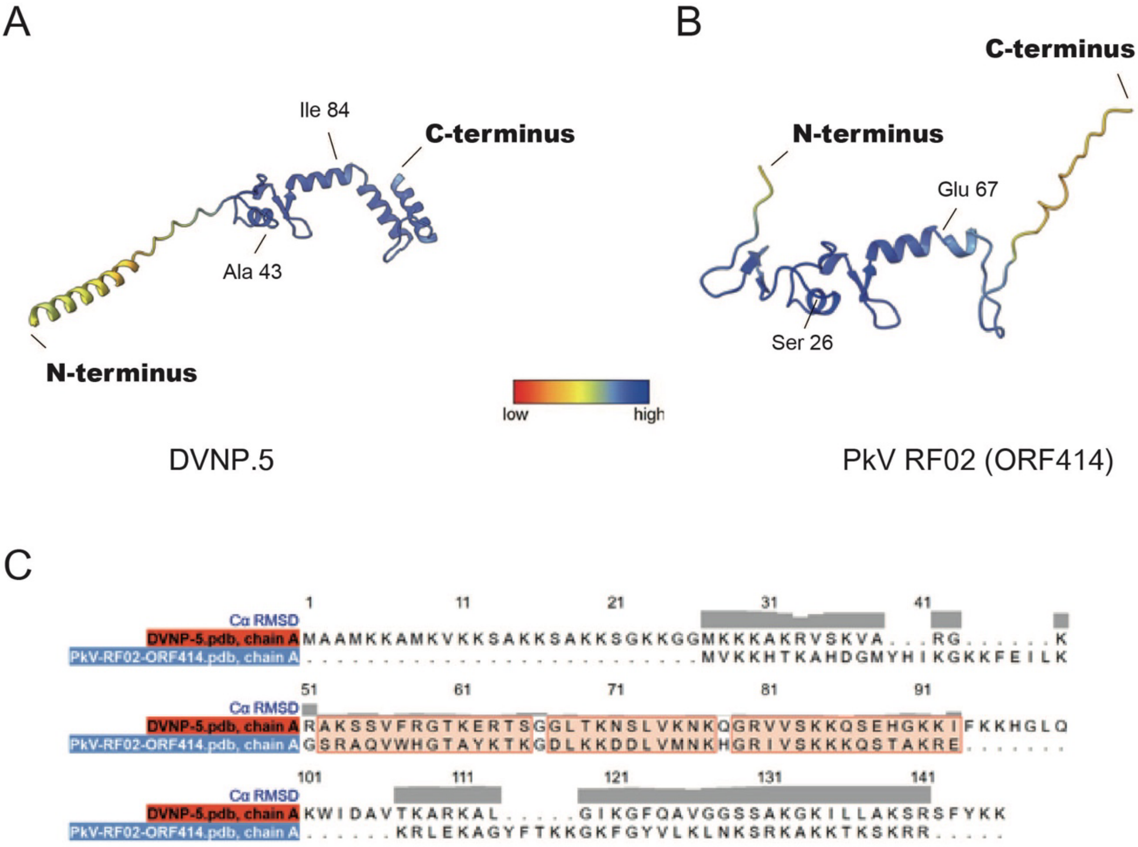

Supplementary Figure 3. Structural prediction and pairwise sequence alignment by Alphafold2.0, coloured by pLDDT confidence measure. A. Prediction of DVNP representative of dinoflagellates DVNP.5. B. Prediction of DVNP representative of *Imitervirales*: PkV RF02 (ORF414). The start and end residues of the structurally aligned regions are marked in A and B. C. Pairwise sequence alignment of Alphafold2.0 predictions of DVNP.5 and PkV RF02 (ORF414), respectively. The structurally aligned amino acids are highlighted in orange.

Supplementary Table 1. Summary of posttranslational modifications encoded by HeV RF02 and PkV RF02

Type	Proteins	Potential Functions	HeV RF02	PkV RF02
**Acetylation**	Acetyltransferase	Chromatin remodelling	ORF59, ORF171, ORF490	ORF68, ORF338, ORF565
**Methylation**	Methyltransferase	Chromatin remodelling	ORF95, ORF400, ORF440, ORF454	ORF501
**Poly(ADP-ribosyl)ation**	Poly [ADP-ribose] polymerase 1-like protein	Regulator of the virus infection cycle	-	ORF73, ORF560
**Prenylation**	Prenyltransferase	Regulator of the virus infection cycle	ORF38, ORF510	ORF365
**Phosphorylation**	Protein kinase, EF-hand-containing protein, IQ-containing protein	Signal transduction	ORF146, ORF203	ORF93, ORF119, ORF157, ORF387, ORF539
**Ubiquitination**	E1, E2, E3 ubiquitin-protein ligase; U-box and intein containing von Willebrand factor type A; RING finger protein; zinc finger protein	Proteolysis, replication activation, a broad range<br><br>of cellular functions.	ORF26, ORF41, ORF73, ORF104, ORF240, ORF280, ORF333, ORF476, ORF507, ORF522	ORF12, ORF19, ORF41, ORF94, ORF116, ORF193, ORF227, ORF247, ORF298, ORF303, ORF422, ORF458, ORF538, ORF595, ORF617, ORF624
**Chaperone**	DnaJ-like protein, heat shock 70kDa protein, cold-shock domain family protein, mitochondrial chaperone BCS1	Protein maturation	ORF29, ORF40, ORF50, ORF105, ORF366, ORF375, ORF418, ORF499, ORF508, ORF519	ORF26, ORF39, ORF59, ORF117, ORF180, ORF200, ORF479, ORF575, ORF597, ORF610
**Protease**	S14	Protein quality control	ORF57, ORF107, ORF126, ORF295, ORF334, ORF344, ORF348, ORF414, ORF429, ORF492	ORF66, ORF134, ORF194, ORF205, ORF213, ORF246, ORF309, ORF398, ORF423, ORF489, ORF567
	C48	SUMO-specific protease		
	metal-dependent hydrolase	DeSUMOylation		
	ATP-dependent protease	Protein disaggregation		
	C1	Proteolysis		
	S74	Virion maturation		
	S16	Degradation of unfolded proteins		
	C19	De-ubiquitination		

Supplementary Table 2. Core genes of the flagellated-protist-infecting viruses

Annotation	Functional categories	Domains organization	HeV-RF02	PkV-RF02	MS (PkV-RF02)[1]
D5-ATPase-helicase	Replication	D5_N, DUF5906, PriCT_2	ORF281	ORF228	-
Proliferating cell nuclear antigen	Replication	PCNA_C, PCNA_N	ORF188	ORF420	-
Topoisomerase type IIA	Replication	DNA_gyraseB, DNA_topoisoIV, Toprim, TOPRIM_C	ORF410	ORF469	-
DNA polymerase B	Replication	DNA_pol_B	ORF328	ORF265	-
DNA mismatch repair protein MutS 7	DNA repair	AAA, MutS_I, MutS_III, MutS_V	ORF160	ORF292	-
Lambda-type exonuclease	DNA recombination	YqaJ	ORF208	ORF331	-
Holliday junction resolvase	DNA recombination	Pox_A22, Ydc2-catalyt	ORF156	ORF167	-
TATA-box binding protein (TBP)	Transcription	TBP	ORF392	ORF410	-
VV D6R-type helicase	Transcription	AAA, DEAD, Helicase_C, ResIII, SNF2-rel_dom	ORF320, ORF129	ORF273	ORF273
RNA polymerase II second largest subunit	Transcription	RNA_pol_Rpb1_1,2,3,4,5,6,7	ORF231, ORF217	ORF285, ORF238	ORF238
Transcription factor TF IIB	Transcription	TFIIB	ORF300	ORF383	-
DNA directed RNA polymerase II largest subunit	Transcription	RNA_pol_Rpb1_1,2,3,4,5,6,7	ORF347	ORF245	ORF245
VLTF-3-like transcription factor	Transcription	Pox_VLTF3	ORF247	ORF314	-
DNA directed RNA polymerase subunit rpb9/M	Transcription	RNA_POL_M_15KD	ORF279	ORF226	ORF226
DNA directed RNA polymerase II subunit rpb3	Transcription	RNA_pol_A_bac, RNA_pol_L, RNA_pol_L_2	ORF260	ORF361	ORF361
DNA-directed RNA polymerase II subunit RPB5	Transcription	RNA_pol_Rpb5_C	ORF288	ORF237	ORF237
DNA directed RNA polymerase K subunit	Transcription	RNA_pol_Rpb6	ORF278	ORF225, ORF274	ORF225, ORF274
Transcription elongation factor TFIIS	Transcription	TFIIS_C, TFIIS_M	ORF277	ORF222	-
Early transcription factor 70 kDa subunit	Transcription	Helicase_C	ORF235	ORF138, ORF204	ORF138, ORF204
VV_A18-like helicase	Transcription	Helicase_C, ResIII,	ORF269	ORF348	-
Major capsid protein MCP	Virus morphogenesis	Capsid_N, Capsid_NCLDV	ORF246	ORF313	ORF313
Major capsid protein MCP2	Virus morphogenesis	Capsid_N, Capsid_NCLDV	ORF199	ORF450	ORF450
A32 virion packaging ATPase	Virus morphogenesis	AAA, Pox_A32	ORF166	ORF460	-
SUMO-1-specific cysteine protease	Posttranslational modification	Peptidase_C48	ORF126	ORF134	-

Metal dependent hydrolase	Posttranslational modification	WLM, YgjP-like	ORF344	ORF213	ORF213
mRNA capping enzyme	Translation	mRNA_cap_enzyme, Pox_MCEL, mRNA_cap_C	ORF316	ORF277	ORF277
mRNA decapping protein	Translation	NUDIX	ORF321	ORF272	-
Ribonucleoside-diphosphate reductase large subunit	Metabolism	Ribonuc_red_lgC, Ribonuc_red_lgN	ORF210	ORF330	-
Ribonucleotide reductase small subunit	Metabolism	Ribonuc_red_sm	ORF175	ORF390	-
Protein disulfide isomerase	Metabolism	Thioredoxin	ORF304, ORF451, ORF98, ORF427	ORF379, ORF487	ORF379
ERV/ALR sulphydryl oxidase	Metabolism	Evr1_Alr	ORF305, ORF322, ORF432	ORF271, ORF492, ORF378	ORF378
Hypothetical protein	Unknown	DUF5762	ORF263	ORF357	ORF357
Hypothetical protein	Unknown	DUF5761	ORF396	ORF404	ORF404
Hypothetical protein	Unknown	DUF5832	ORF169	ORF454	-
Hypothetical protein	Unknown	DUF5767	ORF155	ORF166	-
Hypothetical protein	Unknown	-	ORF172	ORF339	-
Hypothetical protein	Unknown	-	ORF343	ORF214	ORF214

[1] The column of "MS (PkV RF02)" displays the protein-coding genes of PkV RF02 virions detected by mass spectrometry.

Supplementary Table 3. Acronyms

Acronym	Full name
AaV	*Aureococcus anophagefferens* virus
BsV	*Bodo saltans* virus
CeV-01B	*Chrysochromulina ericina* virus
CroV	*Cafeteria roenbergensis* virus
OLPV-1	Organic Lake phycodnavirus 1
OLPV-2	Organic Lake phycodnavirus 2
PgV-16T	*Phaeocystis globosa* virus 16T
TetV1	*Tetraselmis* virus 1
PkV RF01	*Prymnesium kappa* virus RF01
HeV RF02	*Haptolina ericina* virus RF02
PkV RF02	*Prymnesium kappa* virus RF02
EsV	*Ectocarpus siliculosus* virus
FsV	*Feldmannia* sp. Virus
DVNP	Dinoflagellate/viral nucleoprotein
PTMs	Posttranslational modifications
ITRs	Inverted terminal repeats
TEM	Transmission electron microscopy
HGT	Horizontal gene transfer
NCLDVs	Nucleocytoplasmic large DNA viruses
ORFs	Open reading frames
RFC	Replication factor C
PCNA	Proliferating cell nuclear antigen
BER	Base excision repair
MMR	Mismatch repair
ER	Endoplasmic reticulum
3’UTR	3'Untranslated region
eIF4E	Eukaryotic initiation factor 4E
UPR	Unfolded protein response
UDG	Uracil-DNA glycosylase
UvdE	UV damage endonuclease
HJR	Holliday junction resolvase
GTs	Glycosyltransferases
IQR	Interquartile Range
HMMs	Hidden Markov models
GOEV	Global Ocean Eukaryotic Viral database
vMAGs	Viral metagenome-assembled genomes
