# Supplementary material for: Haptophyte-infecting viruses change the genome condensing proteins of dinoflagellates
Source: Commun Biol. 2025 Mar 28;8:510. doi: 10.1038/s42003-025-07905-3 (PMC11953307; doi:10.1038/s42003-025-07905-3)
Supplement: Supplementary file 2 — Description of Additional Supplementary Files [file 42003_2025_7905_MOESM2_ESM.docx]

Description of Additional Supplementary Files

**File name:** Supplementary Data 1

**Description:** Excel spreadsheet containing four sheets, that is, annotation tables for HeV RF02, PkV RF02, tRNA predictions of HeV RF02 and PkV RF02.

**File name:** Supplementary Data 2

**Description:** Detected proteins of mass spectrometry of PkV RF02 virions containing replicates results.

**File name:** Supplementary Data 3

**Description:** Multiple sequence alignment of DVNP homologues.

**File name:** Supplementary Data 4

**Description:** Prediction of NLSs of DVNP homologues.
