# Supplementary material for: Haptophyte-infecting viruses change the genome condensing proteins of dinoflagellates
Source: Commun Biol. 2025 Mar 28;8:510. doi: 10.1038/s42003-025-07905-3 (PMC11953307; doi:10.1038/s42003-025-07905-3)
Supplement: Supplementary file 7 — Reporting summary [file 42003_2025_7905_MOESM7_ESM.pdf]

nature portfolio

Corresponding author(s): Ruth-Anne Sandaa, Haina Wang

Last updated by author(s): Dec 30, 2024

Reporting Summary

Nature Portfolio wishes to improve the reproducibility of the work that we publish. This form provides structure for consistency and transparency in reporting. For further information on Nature Portfolio policies, see our Editorial Policies and the Editorial Policy Checklist.

Statistics

For all statistical analyses, confirm that the following items are present in the figure legend, table legend, main text, or Methods section.

n/a	Confirmed	
☒	☐	The exact sample size (*n*) for each experimental group/condition, given as a discrete number and unit of measurement
☐	☒	A statement on whether measurements were taken from distinct samples or whether the same sample was measured repeatedly
☒	☐	The statistical test(s) used AND whether they are one- or two-sided<br>*Only common tests should be described solely by name; describe more complex techniques in the Methods section.*
☒	☐	A description of all covariates tested
☐	☒	A description of any assumptions or corrections, such as tests of normality and adjustment for multiple comparisons
☒	☐	A full description of the statistical parameters including central tendency (e.g. means) or other basic estimates (e.g. regression coefficient) AND variation (e.g. standard deviation) or associated estimates of uncertainty (e.g. confidence intervals)
☒	☐	For null hypothesis testing, the test statistic (e.g. *F*, *t*, *r*) with confidence intervals, effect sizes, degrees of freedom and *P* value noted<br>*Give P values as exact values whenever suitable.*
☒	☐	For Bayesian analysis, information on the choice of priors and Markov chain Monte Carlo settings
☒	☐	For hierarchical and complex designs, identification of the appropriate level for tests and full reporting of outcomes
☒	☐	Estimates of effect sizes (e.g. Cohen's *d*, Pearson's *r*), indicating how they were calculated

Our web collection on statistics for biologists contains articles on many of the points above.

Software and code

Policy information about availability of computer code

Data collection

GenomeNet Bioinformatics Tools (https://www.genome.jp/)
NCBI non redundant datbase September 2024
Pfam-A
InterPro(https://www.ebi.ac.uk/interpro/)
Global Ocean Eukaryotic Viral database
EcoCyc Database(https://www.ecocyc.org/)
CAZy(https://www.cazy.org/)
MEROPS(https://www.ebi.ac.uk/merops/)
NCLDV database
(https://vogdb.org/; VOGDB release 212)
LC-MS/MS
Transmission electron microscopy (TEM)

Data analysis

blast+
PSI-BLAST v2.10.1
hmmsearch v3.2.1
MAFFT - linsi v7.505
Trimal1.4.1
IQ-tree v.1.6.2
Fasttree v2
iTOL(https://itol.embl.de/)

GeneMarkS
tRNAscan(http://lowelab.ucsc.edu/tRNAscan-SE/)
SignalP6
MUMmer package
DeepTMHMM
AlphaFold2
UCSF ChimeraX

For manuscripts utilizing custom algorithms or software that are central to the research but not yet described in published literature, software must be made available to editors and reviewers. We strongly encourage code deposition in a community repository (e.g. GitHub). See the Nature Portfolio guidelines for submitting code & software for further information.

Data

Policy information about availability of data

All manuscripts must include a data availability statement. This statement should provide the following information, where applicable:

- Accession codes, unique identifiers, or web links for publicly available datasets
- A description of any restrictions on data availability
- For clinical datasets or third party data, please ensure that the statement adheres to our policy

The nucleotide sequences will be deposited and available in GenBank before publication. The data of LC-MS/MS will be deposited in public database before publication.

Research involving human participants, their data, or biological material

Policy information about studies with human participants or human data. See also policy information about sex, gender (identity/presentation), and sexual orientation and race, ethnicity and racism.

Reporting on sex and gender	NA.
Reporting on race, ethnicity, or other socially relevant groupings	NA.
Population characteristics	NA.
Recruitment	NA.
Ethics oversight	NA.

Note that full information on the approval of the study protocol must also be provided in the manuscript.

Field-specific reporting

Please select the one below that is the best fit for your research. If you are not sure, read the appropriate sections before making your selection.

☐ Life sciences ☐ Behavioural & social sciences ☒ Ecological, evolutionary & environmental sciences

For a reference copy of the document with all sections, see nature.com/documents/nr-reporting-summary-flat.pdf

Ecological, evolutionary & environmental sciences study design

All studies must disclose on these points even when the disclosure is negative.

Study description	Morphological and molecular analyses of two marine giant viruses infecting haptophytes.
Research sample	Haptolina ericina virus RF02 (HeV RF02) and Prymnesium kappa virus RF02 (OkV RF02) assigned to Mesomimiviridae.
Sampling strategy	The samples for TEM observation were collected during infection cycle with three parallels. The samples for LC-MS/MS were collected from different infections and tested for twice independently.
Data collection	The sequence data was collected by Haina Wang, Lingjie Meng, Romain Blanc-Mathieu and Håkon Dahle from 2019 to 2024. The data of LC-MS/MS was collected by Haina Wang, Sara Otaegi-Ugartemendia and Gabriela N. Condezo in 2023. TEM images were collected by Haina Wang from 2020 to 2023.
Timing and spatial scale	Sequence data was collected from 2019-2024 by Haina Wang, Håkon Dahle, David Brand and Jörn Kalinowski.

Data exclusions	There is no unpublished data in this study.
Reproducibility	The experiment of LC-MS/MS has been reproduced. The other experiments including TEM observation and data analysis has not been reproduced but can be reproduced with the methods outlined in the manuscript.
Randomization	Randomization is not relevant in this study as all available data has been collected for NCLDVs related analyses.
Blinding	Blinding was not relevant for this study as the same methods have been applied to the entire datasets.

Did the study involve field work? ☐ Yes ☒ No

Reporting for specific materials, systems and methods

We require information from authors about some types of materials, experimental systems and methods used in many studies. Here, indicate whether each material, system or method listed is relevant to your study. If you are not sure if a list item applies to your research, read the appropriate section before selecting a response.

Materials & experimental systems

n/a	Involved in the study
☒	☐ Antibodies
☒	☐ Eukaryotic cell lines
☒	☐ Palaeontology and archaeology
☒	☐ Animals and other organisms
☒	☐ Clinical data
☒	☐ Dual use research of concern
☒	☐ Plants

Methods

n/a	Involved in the study
☒	☐ ChIP-seq
☒	☐ Flow cytometry
☒	☐ MRI-based neuroimaging

Plants

Seed stocks	NA.
Novel plant genotypes	NA.
Authentication	NA.
